# Prophylactic Beta-Blocker Therapy in Patients Who Underwent Primary Percutaneous Coronary Intervention for ST-Elevation Myocardial Infarction With Preserved Left Ventricular Ejection Fraction: A Systematic Review and Meta-Analysis

**DOI:** 10.7759/cureus.98992

**Published:** 2025-12-11

**Authors:** Ziad Affas, Keyur Patel, Omr R Abuzahrieh, Saman Al Barznji, Mohammed Walji, Rowaid Touza, Manar Albanna, Marcel Zughaib

**Affiliations:** 1 Internal Medicine, Henry Ford Health Providence, Southfield, USA; 2 Internal Medicine, Henry Ford Macomb Hospital, Clinton Township, USA; 3 Internal Medicine, McLaren Health Care, Pontiac, USA; 4 Internal Medicine, Hawler Medical University, Erbil, IRQ

**Keywords:** all-cause mortality, a systematic review, beta-blockers, cardiovascular mortality, heart failure hospitalization, meta-analysis, percutaneous coronary intervention (pci), preserved ejection fraction, reinfarction, st-elevation myocardial infarction (stemi)

## Abstract

The role of prophylactic beta-blocker therapy in patients treated with primary percutaneous coronary intervention (PCI) for ST-elevation myocardial infarction (STEMI) who have mildly reduced or preserved left ventricular ejection fraction (LVEF) remains a subject of debate. This systematic review and meta-analysis aimed to evaluate the efficacy of beta-blocker therapy versus no beta-blocker therapy in this specific patient population. We searched PubMed, National Institutes of Health (NIH), Elsevier, Google Scholar, and ClinicalTrials.gov for studies published between 2014 and 2024. Eligible studies included randomized controlled trials (RCTs) and observational studies comparing beta-blockers with no beta-blockers in patients undergoing PCI for STEMI with an LVEF ≥ 40%. The primary outcome was all-cause mortality; secondary outcomes included cardiovascular mortality, reinfarction, and hospitalization for heart failure or stroke. Effect sizes were calculated as relative risk (RR) with 95% confidence intervals (CI).

Our search yielded 187 articles, from which six studies (four observational and two RCTs) met the inclusion criteria, encompassing a pooled cohort of 28,736 patients (mean age: 63.5 years; 83% male). Of these, 13,650 (47.5%) received beta-blockers at hospital discharge. The meta-analysis of five studies (18,459 patients) indicated that beta-blocker use was associated with a significant reduction in all-cause mortality. However, heterogeneity was high; sensitivity analysis removing one influential RCT reduced I² to 55% and strengthened the significance. Subgroup analysis for cardiovascular mortality (four studies, 14,784 patients) showed a significant risk reduction in two observational studies but no significant effect in two RCTs. No significant differences were observed for reinfarction or hospitalization for heart failure or stroke. The quality of evidence from RCTs was deemed low due to their open-label design. In conclusion, while beta-blocker therapy may reduce all-cause mortality in post-PCI STEMI patients with preserved LVEF, its effect on cardiovascular mortality is inconsistent between study types. High-quality, blinded RCTs are warranted to definitively establish efficacy in this population.

## Introduction and background

Long-term oral beta-blocker therapy is a class I recommendation by both the American Heart Association/American College of Cardiology (AHA/ACC) and the European Society of Cardiology (ESC) for patients who develop heart failure, left ventricular (LV) dysfunction, or ventricular tachyarrhythmias after ST-elevation myocardial infarction (STEMI) [1,2]. These recommendations are historically grounded in numerous studies demonstrating beta-blocker efficacy [1]. However, a significant portion of this foundational evidence predates the widespread adoption of primary percutaneous coronary intervention (PCI) as the standard reperfusion strategy for STEMI, a development that has substantially altered the therapeutic landscape and patient outcomes [3,4]. Despite this evolution, beta-blockers remain standard care for post-STEMI patients with cardiac dysfunction [1,2].

Conversely, the prophylactic use of beta-blockers, defined as the routine initiation of therapy in STEMI patients without heart failure, LV dysfunction, or ventricular arrhythmias, presents a more nuanced picture. The AHA/ACC guidelines maintain a class I recommendation for beta-blocker therapy in this broader post-MI population, whereas the ESC guidelines adopt a more conservative class IIa recommendation, signaling uncertainty [1,2]. This divergence highlights an ongoing debate that stems from different interpretations of available evidence in the contemporary PCI era. The clinical relevance of this distinction is significant: A class I recommendation implies that a treatment should be administered based on strong evidence, while a class IIa recommendation suggests the benefit is less certain and treatment may be considered. This debate has been further fueled by recent studies questioning the mortality benefits of beta-blockers in STEMI patients with normal ejection fraction (EF) in the modern PCI era, including a large, contemporary randomized controlled trial (RCT) [5].

Given these evolving perspectives and advancements in STEMI management, a re-evaluation of long-term oral beta-blocker therapy is warranted, particularly for patients with preserved LV function post-PCI. This is crucial because while historical data showed clear benefits, modern PCI has dramatically improved baseline outcomes, thus questioning the incremental value of prophylactic therapies. This systematic review and meta-analysis aims to synthesize recent evidence on the utility of prophylactic beta-blockers in patients with STEMI and preserved LVEF who have undergone primary PCI. Our objective is to assess the impact of beta-blockers on all-cause mortality, cardiovascular mortality, reinfarction, and hospitalization for heart failure or stroke in this specific cohort. A clearer understanding of beta-blocker effects in this contemporary setting is crucial for optimizing patient care, avoiding potential overuse, and identifying gaps in the existing literature, such as the limited availability of high-quality RCTs, that warrant future research.

## Review

Methods

This systematic review and meta-analysis were conducted in accordance with the PICOS (Population, Intervention, Comparison, Outcome, Study Design) framework [6] and are reported in accordance with the Preferred Reporting Items for Systematic Reviews and Meta-Analyses (PRISMA) 2020 statement [7]. A comprehensive search was performed across PubMed/MEDLINE, ClinicalTrials.gov, Elsevier/Science databases, and Google Scholar for studies published between January 1, 2014, and December 31, 2024, following PICOS criteria. Both Medical Subject Headings (MeSH) and free-text terms were used, combined with Boolean operators (AND/OR). The primary keywords included “beta-blocker,” “myocardial infarction,” and “heart failure with preserved ejection fraction (HFpEF).” Searches were adapted for each database using the Polyglot Search Translator, and duplicates were removed using the Systematic Review Accelerator (SRA). We limited results to human studies published in English. The review followed PRISMA guidelines, and the screening process was independently performed by two reviewers, with disagreements resolved through consensus and third-party adjudication. Analyses were conducted using available data only, and no imputation was performed. The PICOS elements are detailed in Table 1.

**Table 1:** PICOS criteria This table outlines the PICOS (Population, Intervention, Comparison, Outcome, Study Design) framework that was used to define the eligibility criteria for studies included in this systematic review and meta-analysis. Source: Adopted from Thomas et al. (2019) [6].

Population	Patients post-myocardial infarction or acute coronary syndrome who underwent coronary angiography with preserved left ventricular ejection fraction ≥ 40%
Intervention	Beta-blocker
Control	No beta-blocker
Outcome	Primary outcomes: All-cause mortality
	Secondary outcomes: Death due to cardiovascular causes, reinfarction, hospitalization due to heart failure, and hospitalization due to stroke
Study design	Retrospective observational study, randomized control trial, and prospective observational study

Selection Criteria

Original comparative studies (RCTs, prospective or retrospective observational cohort studies) published in English between January 2014 and April 2024 were considered. Studies were included if they evaluated prophylactic oral beta-blocker therapy versus no beta-blocker therapy in adult patients who underwent primary PCI for STEMI and had a preserved LVEF (defined as ≥40%). Studies were excluded if patients had a clear clinical indication for or contraindication to beta-blocker therapy, died during the index hospitalization, or had a terminal illness at discharge. Detailed inclusion and exclusion criteria are presented in Table 2.

**Table 2:** Selection criteria for included studies This table details the specific inclusion and exclusion criteria applied during the screening process, covering language, publication timeframe, type of studies, geographical region, and target patient population.

Criteria	Inclusion	Exclusion
Language	English	All other languages
Timeframe of publication	2014–April 2024	Older publications
Type of studies	Comparative original articles: retrospective observational studies, prospective observational studies, randomized controlled trials	Case reports, case series, protocols, reviews, gray literature
Region	All	-
Target population	Patient who underwent coronary angiography post-myocardial infarction/acute coronary syndrome with preserved left ventricular ejection fraction (LVEF) ≥ 40%	Patients with LVEF < 40%, clinical indication or contraindication to beta-blocker use, in-hospital death, terminal illness at the time of discharge

Search Strategy

We conducted a systematic search of PubMed, NIH, Elsevier, Google Scholar, and ClinicalTrials.gov. The search string combined MeSH, such as beta-blocker, myocardial infarction (MI), and HFpEF. Boolean operators (AND, OR) were used appropriately. The Cochrane Database search manager was consulted to identify additional relevant MeSH terms. The Polyglot Search Translator was used to adapt the search string for different databases, minimizing translation bias [8]. Filters for publication year (2014-2024) and study design (RCTs and observational studies) were applied where available.

Duplicates were removed using the SRA tool [9] and manual checks. Two investigators independently screened titles and abstracts. Full-text articles of potentially eligible studies were retrieved and independently assessed for eligibility by two authors based on the predefined selection criteria as outlined in Table 2. Any disagreements were resolved by discussion or consultation with a third author. Reasons for excluding studies at the full-text stage were documented. Publications meeting predefined PICO criteria and reporting raw data for outcome variables were considered qualified for final analysis; studies reporting data in the form of ratios without raw original data were excluded. Additionally, reference lists of included studies and relevant systematic reviews were manually searched for potentially eligible publications.

Data Extraction

Two authors independently extracted data from included studies. Extracted information included first author, publication year, study design, sample size, patient characteristics (mean/median age, sex distribution, and LVEF criteria), specific beta-blocker(s) used, and duration of follow-up. The data were cross-checked by the authors, compiled for evaluation of intervention and outcome, and summarized in Table 3.

Quality Assessment

The quality of included observational studies was assessed using the Newcastle-Ottawa Scale (NOS) [10], which evaluates selection, comparability, and outcome assessment. The risk of bias was assessed independently by two reviewers using the NOS for observational studies and the Cochrane Risk of Bias-2 (RoB-2) tool for RCTs. Discrepancies were resolved by discussion and, when necessary, by a third reviewer. Observational studies generally lost points in the selection and comparability domains due to incomplete adjustment for potential confounders or limited reporting of follow-up completeness. Both included RCTs were open-label and therefore judged as having high risk in the performance and detection domains. Studies were scored out of a maximum of nine stars, with higher scores indicating a lower risk of bias. RCTs were assessed using the Cochrane RoB-2 tool [11], which evaluates bias arising from the randomization process, deviations from intended interventions, missing outcome data, measurement of the outcome, and selection of the reported result. Each domain was judged as "low risk of bias," "some concerns," or "high risk of bias." Publication bias was not formally assessed using funnel plots due to the small number of included studies (<10) for any single outcome, as recommended [12]. However, a search of the reference lists of included articles and relevant reviews was conducted to identify potentially missed studies.

Quantitative Analysis (Meta-analysis)

Statistical analyses were conducted using RevMan 5.4 (The Cochrane Collaboration, London, England, UK), employing random-effects models (DerSimonian-Laird method) to account for both within- and between-study variability. Effect estimates were expressed as relative risks (RRs) with 95% confidence intervals (CIs). Studies were weighted by the inverse variance method, incorporating both within-study precision and between-study variance. Risk difference values reported in the draft were corrected to reflect RR reductions for clarity. Statistical heterogeneity was evaluated using the I² statistic and interpreted as low (<30%), moderate (30%-60%), or high (>60%). Outcomes with I² > 50% underwent leave-one-out sensitivity analyses, in which an individual study was considered influential if its exclusion altered the pooled estimate by >10%, changed statistical significance, or reduced I² by ≥15%. Subgroup analyses were prespecified by study design (RCT vs observational). CIs and P-values are reported consistently across all pooled outcomes. Publication bias was not formally tested using funnel plots or Egger’s regression because fewer than 10 studies were available per outcome.

Results

Eligible Studies and Study Selection

**Figure 1:**
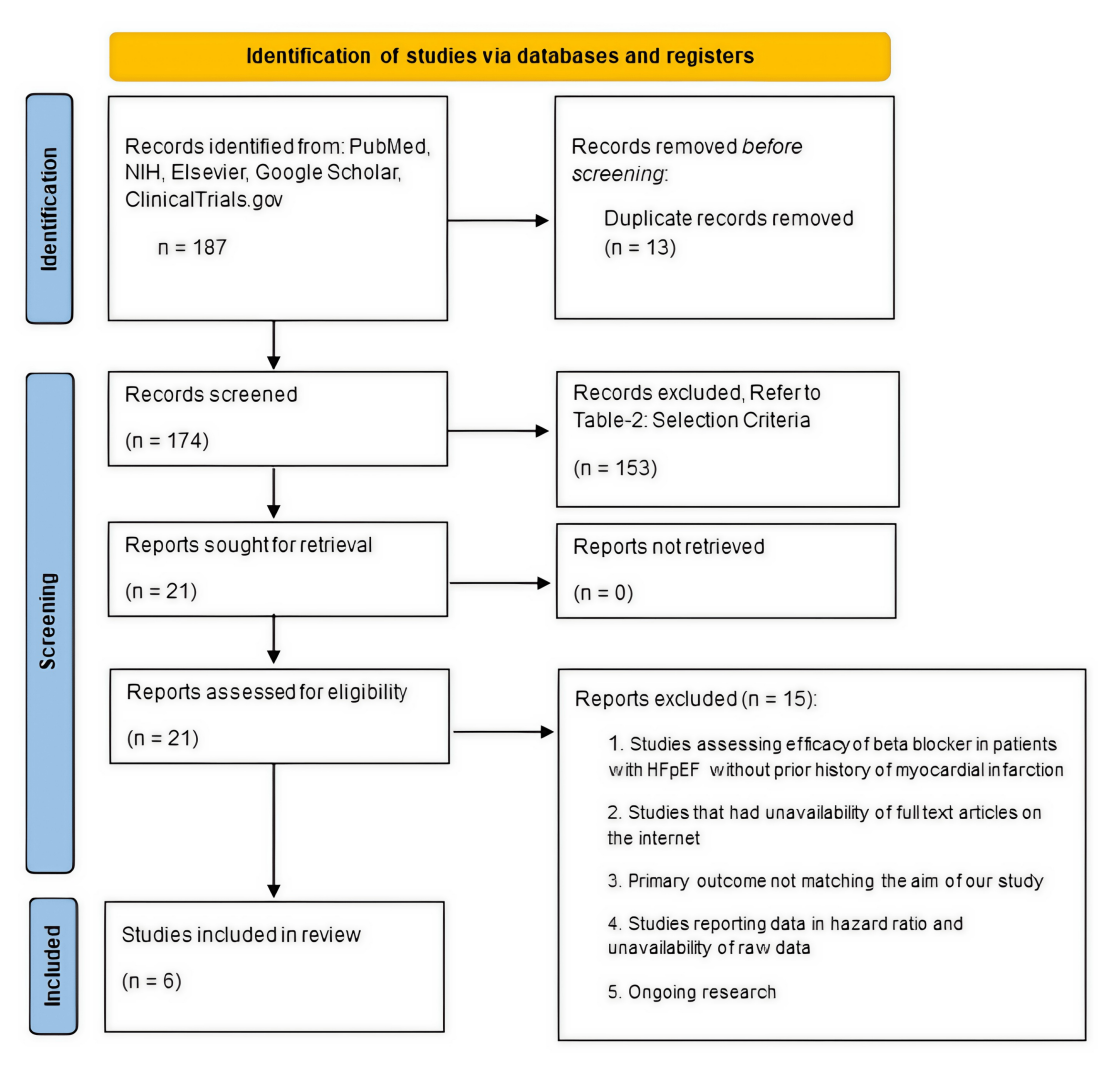
PRISMA 2020 flow diagram for study selection The diagram illustrates the flow of information through the different phases of the systematic review. It outlines the number of records identified, included, and excluded, as well as the reasons for exclusion at each stage—from the initial database search yielding 187 articles to the final six studies included in the meta-analysis. HFpEF: Heart failure with preserved ejection fraction.

The initial search yielded 187 articles. After removing 13 duplicates, 174 unique records were screened by title and abstract. This screening process led to the exclusion of 153 articles that did not meet the initial criteria (e.g., wrong population, intervention, study design, or studies that were not original research). The full texts of the remaining 21 articles were assessed for eligibility. Of these, 15 were excluded for reasons such as LVEF < 40% or not specified, no PCI population, no control group without beta-blockers, outcomes not relevant or raw data unavailable, or ongoing trials. Ultimately, six studies met all inclusion criteria and were included in this systematic review and meta-analysis [5,13-17]. The PRISMA flow diagram detailing the study selection process is presented in Figure 1.

Study Characteristics

The six included studies comprised two RCTs [5,14] and four observational cohort studies [13,15-17]. These studies were published between 2014 and 2024 and collectively enrolled 28,736 patients. Of these, 13,650 (47.5%) received beta-blocker therapy at hospital discharge, and 15,086 (52.5%) did not. The mean age of the pooled patient population was approximately 63.5 years, and the majority of patients were male (ranging from 75% to 83% across studies). Specific beta-blockers varied: One study used carvedilol exclusively [14], one used either carvedilol or metoprolol [16], and one used bisoprolol or metoprolol [5]. The remaining three observational studies did not specify the type of beta-blocker used [13,15,17]. The mean or median follow-up duration ranged from one year [15,17] to 4.5 years [16]. Key characteristics of the included studies are summarized in Table 3.

**Table 3:** Baseline characteristics of included studies This table summarizes the key characteristics of the six studies included in the meta-analysis. Data presented include the first author and publication year, study design, sample size, patient population characteristics, demographics, intervention and control group sizes, randomization process (for RCTs), and the mean or median follow-up duration. LVEF: Left ventricular ejection fraction; STEMI: ST-elevation myocardial infarction; PCI: Percutaneous coronary intervention; HF: Heart failure; BB: Beta-blocker; MI: Myocardial infarction.

Author, Year	Study Design	Sample Size	Population Characteristics	Mean Age (IQ range)	Male Gender	Female Gender	Intervention Beta-Blocker Group	Control No Beta-Blocker Group	Randomization Process	Median/Mean Follow-Up (IQ Range)
Yndigegn et al. (2024) [5]	Parallel-group, open-label randomized clinical trial	5020	1 to 7 days post-myocardial infarction who had undergone coronary angiography with preserved LVEF	65 years	77.5%	22.5%	2508	2512	1:1	3.5 years (2.2-4.7)
López et al. (2020) [13]	Retrospective observational study	460	Patients having STEMI in the presence of atherosclerotic plaque without HF (LVEF ≥ 40%)	63.8 years (±13.8)	75.7%	24.3%	356	104	-	Mean 3.9 years (2.6-5.45)
Watanabe et al. (2018) [14]	Open-label, randomized controlled trial	794	Patients having PCI after STEMI with preserved LVEF ≥ 40%	64 (±12) years	83% (BB), 78% (No BB)	17% (BB), 22% (No BB)	394	400	1:1	Mean 3.9 years
D’Ascenzo et al. (2018) [15]	Multicenter retrospective study	5870	Patients with acute coronary syndromes (ACS) treated with PCI and without HF/preserved LVEF	66 years	75%	25%	2935	2935	-	1 year
Lee et al. (2015) [16]	Retrospective cohort study	901	Clinical outcomes in patients post-MI treated with PCI with preserved LVEF	58 years	80%	20%	598	303	-	4.5 years
Yang et al. (2014) [17]	Prospective e-cohort study	8510	Clinical outcomes in patients post-MI treated with PCI with preserved LVEF	63 years	75%	25%	6873	1637	-	1 year

Quality assessment

The quality assessment of the four observational studies using NOS indicated a generally low risk of bias. Most studies scored seven to nine stars, demonstrating adequate selection of cohorts, comparability, and outcome ascertainment (Table 4).

**Table 4:** Quality assessment of observational studies using Newcastle-Ottawa scale (a) Case definition OR adequate representativeness of the exposed cohort; (b) representativeness of the cases OR selection of the non-exposed cohort; (c) selection of controls OR ascertainment of exposure; (d) definition of controls OR demonstration that the outcome of interest was not present at the start of the study; (e) comparability of cases and controls based on the design or analysis OR comparability of cohorts based on the design or analysis; (f) ascertainment of exposure OR assessment of outcome; (g) same method of ascertainment for cases and controls OR follow-up long enough for outcomes to occur; and (h) non-response rate OR adequacy of follow-up of cohorts. When there is selective grouping, the average or median follow-up time is less than six months, or the follow-up rate is less than 90%, corresponding to the representativeness of the exposed cohort, follow-up long enough for outcomes, or adequacy of follow-up receives 0 points. When adding up the scores and the total mark is 9 points, 8–9 points indicate low risk of bias; 6–7 points indicate moderate risk of bias; 1–5 points indicate high risk of bias; and 0 points indicate very high risk of bias.

New-Castle Ottawa Scale [10]									
Cohort Study	Selection			Comparability			Outcome		Total
	1a	2b	3c	4d	5e	6f	7g	8h	
Yang et al. 2014 [14]	1	1	0	1	1	1	0	1	6
Lee et al. 2015 [13]	1	1	0	1	1	1	1	1	7
D’Ascenzo et al. (2018) [15]	1	1	1	1	1	1	0	1	7
López et al. (2020) [13]	1	1	1	1	1	1	1	1	8

**Figure 2:**
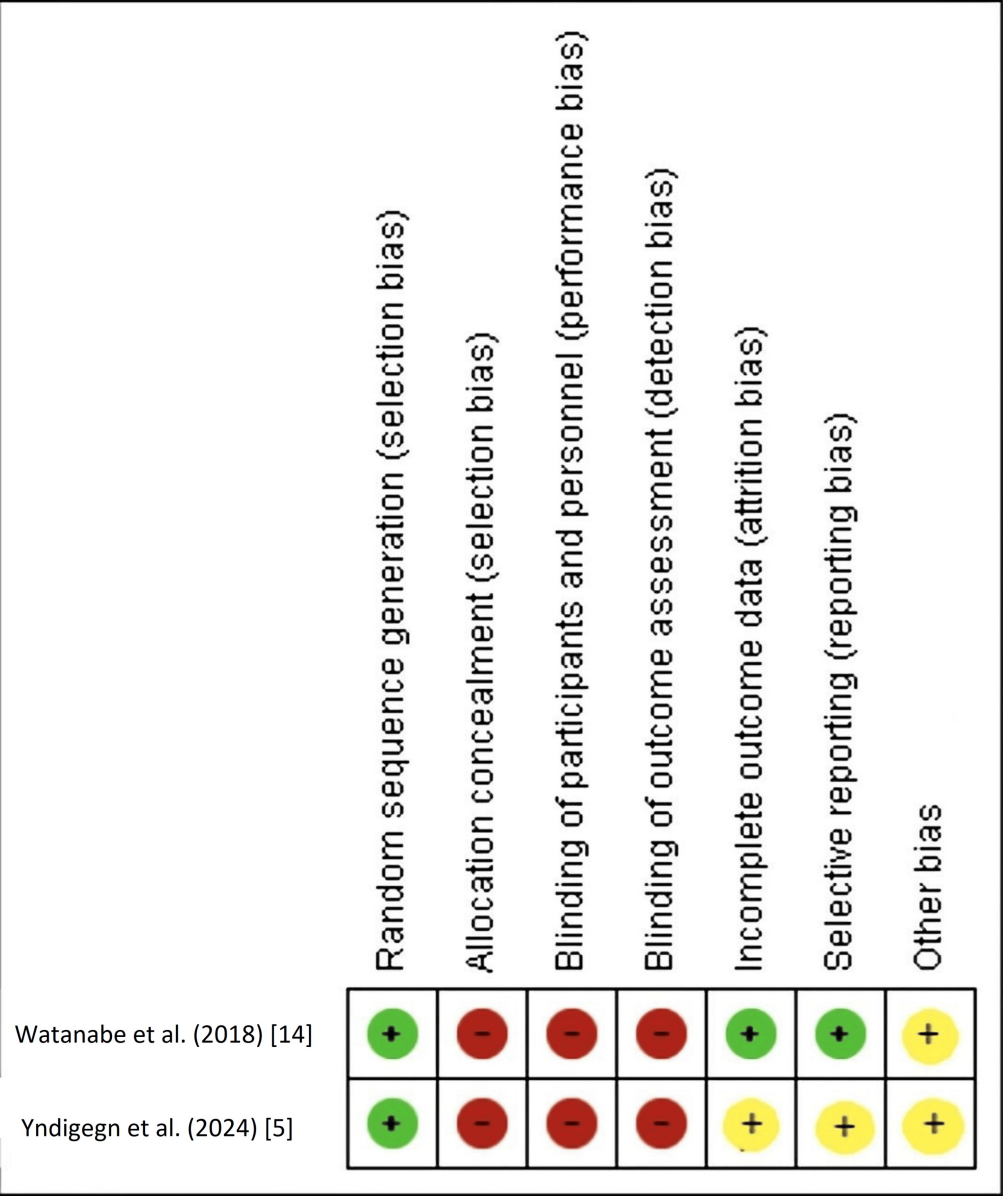
Risk of bias summary for randomized controlled trials This figure provides a study-by-study summary of the risk of bias assessment for the two included RCTs (Watanabe et al., 2018 [14], and Yndigegn et al., 2024 [5]), based on the Cochrane Risk of Bias-2 (RoB-2) tool. Each row represents an individual study, and each column corresponds to a specific bias domain. The color and symbol in each cell indicate the judgment for that study and domain: green (+) for low risk of bias, yellow for some concerns, and red (–) for high risk of bias.

**Figure 3:**
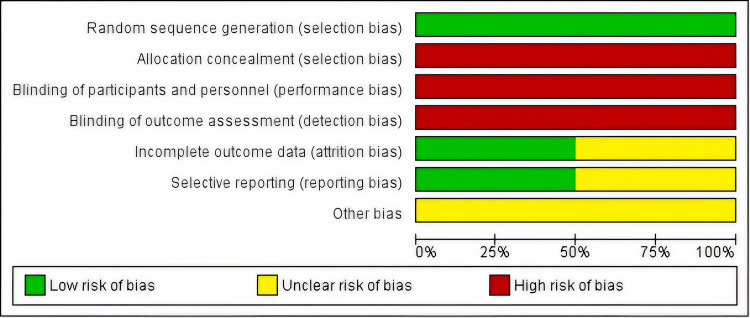
Risk of bias graph for randomized controlled trials This graph visualizes the aggregated risk of bias judgments across the two included RCTs for each domain of the Cochrane RoB-2 tool. Each bar represents a bias domain, and the colored segments indicate the proportion of studies judged to be at low risk of bias (green), some concerns (yellow), or high risk of bias (red). The figure highlights that the primary sources of potential bias were related to performance bias and detection bias, stemming from the open-label design of the trials. RCT: Randomized controlled trial; RoB-2: Risk of Bias-2.

For the two RCTs, the Cochrane RoB-2 tool revealed "some concerns" or "high risk of bias" primarily related to deviations from intended interventions (due to open-label design) and potential bias in measurement of the outcome (performance and detection bias, as neither participants nor clinicians were blinded). The randomization process and risk of missing outcome data were generally judged as low risk or some concerns. Detailed RoB-2 assessments are presented in Figure 2 (summary) and Figure 3 (graph).

Primary Outcome: All-Cause Mortality

**Figure 4:**
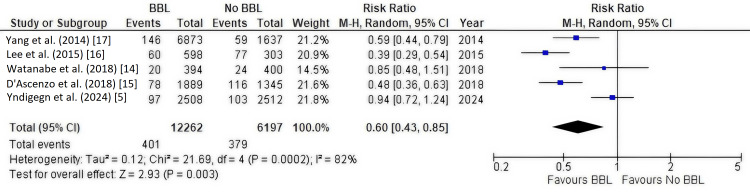
Forest plot of all-cause mortality This forest plot visualizes the meta-analysis of the primary outcome, all-cause mortality. Each square represents the relative risk (RR) from an individual study, with the horizontal line indicating the 95% confidence interval (CI). The size of the square is proportional to the study's weight in the analysis. The diamond represents the pooled RR estimate, which shows a statistically significant reduction in all-cause mortality with beta-blocker therapy (RR, 0.60; 95% CI, 0.43-0.85). The I² statistic indicates substantial heterogeneity between studies. BBL: Beta-blockers.

Five studies [5,14-17] involving 18,459 patients reported data on all-cause mortality. The pooled analysis showed that beta-blocker therapy was associated with a statistically significant 40% reduction in the risk of all-cause mortality compared to no beta-blocker therapy (RR, 0.60; 95% CI, 0.43-0.85; P = 0.003). However, there was substantial heterogeneity among the studies (I² = 82%) (Figure 4).

In a sensitivity analysis, removal of the Yndigegn et al. (2024) study [5], an RCT that found no benefit for beta-blockers, decreased heterogeneity (I² = 55%) and enhanced the statistical significance of the pooled estimate for all-cause mortality with beta-blockers (P < 0.00001).

Secondary Outcome: Cardiovascular Mortality

**Figure 5:**
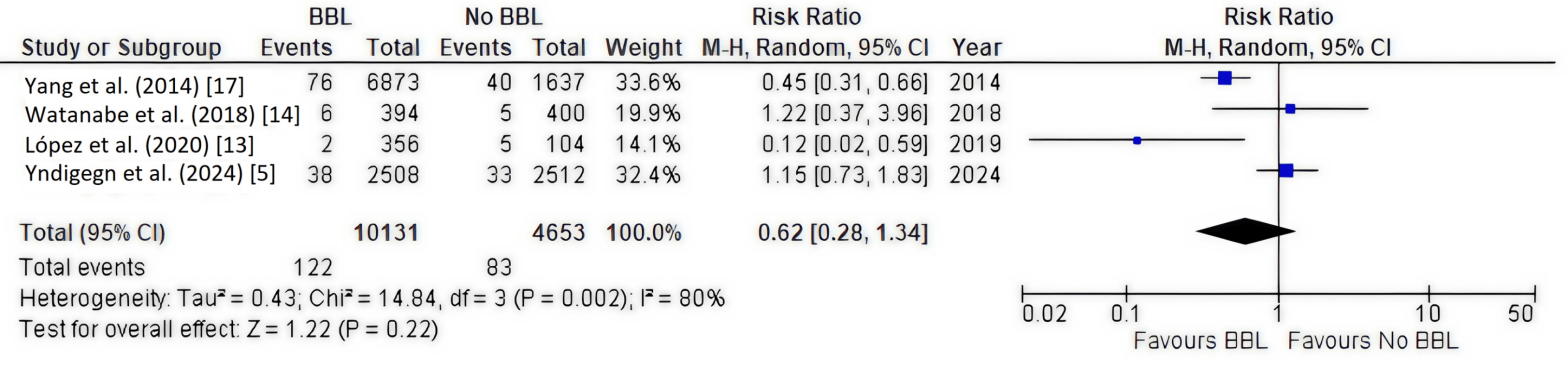
Forest plot of cardiovascular mortality (all studies) This forest plot shows the pooled analysis for the secondary outcome of cardiovascular mortality across all included study types. The diamond represents the overall relative risk (RR), which did not show a statistically significant difference between the beta-blocker and no-beta-blocker groups (RR, 0.62; 95% CI, 0.28-1.34). The I² statistic indicates high heterogeneity. BBL: Beta-blockers.

**Figure 6:**
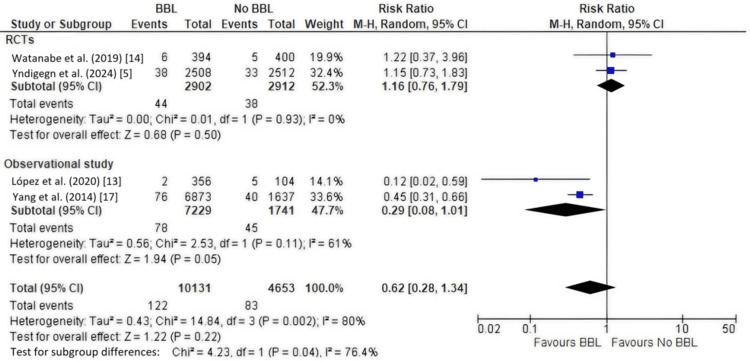
Forest plot of cardiovascular mortality (subgroup analysis by study design) This plot presents a subgroup analysis for cardiovascular mortality, separating randomized controlled trials (RCTs) and observational studies. The results show a trend toward benefit in observational studies (RR, 0.29; 95% CI, 0.08-1.01) but no effect in RCTs (RR, 1.16; 95% CI, 0.76-1.79). The test for subgroup differences indicates a significant discrepancy between the study types. BBL: Beta-blockers.

Four studies [5,13,14,17], including 14,784 patients, reported cardiovascular mortality. The overall pooled analysis did not show a significant difference between the beta-blocker and no beta-blocker groups (RR, 0.62; 95% CI, 0.28-1.34; P = 0.22), with high heterogeneity (I² = 80%) (Figure 5), which did not decrease with sensitivity leave-one-out analysis. Subgroup analysis by study design was performed (Figure 6).

Observational studies (two studies) [13,17]: Beta-blocker use was associated with a trend toward significantly lower cardiovascular mortality (RR, 0.29; 95% CI, 0.08-1.01; P = 0.05), with moderate heterogeneity (I² = 61%).

RCTs (two studies) [5,14]: Beta-blocker use showed no significant effect on cardiovascular mortality (RR, 1.16; 95% CI, 0.76-1.79; P = 0.50), with no heterogeneity (I² = 0%).

Secondary Outcome: Reinfarction

**Figure 7:**
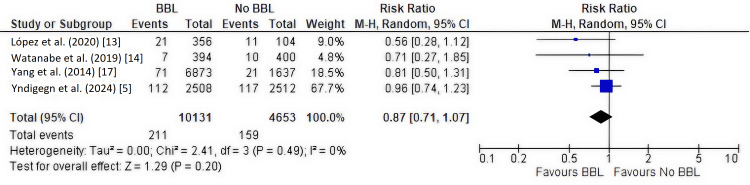
Forest plot of reinfarction This forest plot visualizes the meta-analysis for the secondary outcome of reinfarction. The pooled relative risk (RR), represented by the diamond, shows no significant difference between the beta-blocker and no beta-blocker groups (RR, 0.87; 95% CI, 0.71-1.07). BBL: Beta-blockers.

Four studies [5,13,14,17], including 14,784 patients, assessed the incidence of reinfarction. There was no significant difference in reinfarction rates between the beta-blocker and no beta-blocker groups (RR, 0.87; 95% CI, 0.71-1.07; P = 0.20), with no heterogeneity (I² = 0%) (Figure 7).

Secondary Outcome: Hospitalization for Heart Failure and Stroke

Hospitalization due to heart failure and stroke was reported by the two RCTs [5,14], including 5,814 patients.

**Figure 8:**
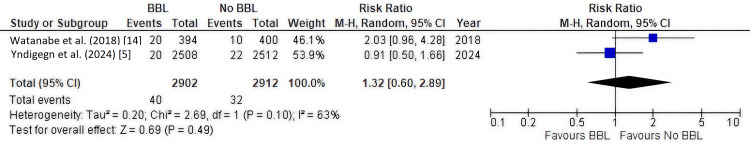
Forest plot of hospitalization for heart failure This plot shows the meta-analysis for hospitalization for heart failure from the two included randomized controlled trials (RCTs). The pooled relative risk (RR, 1.32; 95% CI, 0.60-2.89) indicates no significant difference between the groups. BBL: Beta-blockers.

Hospitalization for heart failure: There was no significant difference between groups (RR, 1.32; 95% CI, 0.60-2.89; P = 0.49), with moderate heterogeneity (I² = 63%) (Figure 8).

**Figure 9:**
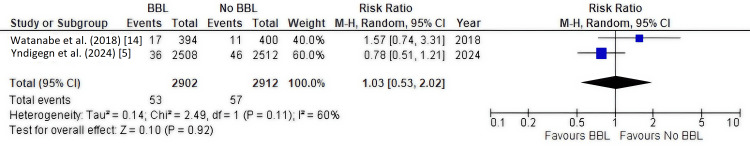
Forest plot of hospitalization for stroke This plot shows the meta-analysis for hospitalization for stroke from the two included randomized controlled trials (RCTs). The pooled relative risk (RR, 1.03; 95% CI, 0.53-2.02) indicates no significant difference between the groups. BBL: Beta-blockers.

Hospitalization for stroke: There was no significant difference between groups (RR, 1.03; 95% CI, 0.53-2.02; P = 0.92), with moderate heterogeneity (I² = 60%) (Figure 9).

Discussion

This systematic review and meta-analysis evaluated the role of prophylactic beta-blocker therapy in patients with STEMI and preserved LVEF (≥40%) who underwent primary PCI. Our main finding suggests that beta-blocker use at discharge may be associated with a reduction in all-cause mortality. However, this result was characterized by high heterogeneity, primarily driven by conflicting results between observational studies and RCTs, particularly the recent REDUCE-AMI (Randomized Evaluation of Decreased Usage of Beta-Blockers after Acute Myocardial Infarction) trial [5]. When the REDUCE-AMI trial was excluded in a sensitivity analysis, the observed benefit for all-cause mortality became more consistent, and heterogeneity decreased. This finding, combined with our formal subgroup analysis for cardiovascular mortality, which found a statistically significant difference between the outcomes of observational studies and RCTs (P = 0.04), provides strong evidence that the primary source of heterogeneity is the conflicting results between different study designs. This underscores that the central issue is one of evidence quality and study methodology.

Beta-blockers are traditionally thought to confer benefits post-MI by reducing myocardial oxygen demand (MVO₂) through decreases in heart rate, blood pressure, and contractility, thereby mitigating ischemia, minimizing ventricular arrhythmias, and improving long-term survival [18]. They may also favorably influence cardiac bioenergetics and attenuate adverse remodeling post-infarction [19,20]. Current AHA/ACC guidelines recommend beta-blockers for all post-MI patients, while ESC guidelines are more reserved for those without LV dysfunction [1,2]. Our findings, which show a mortality benefit driven by observational data but not supported by contemporary RCTs, lend more weight to the conservative approach of the ESC guidelines. The lack of clear benefit in RCTs suggests that downgrading the broad class I recommendation from the AHA/ACC for this specific patient population may be warranted pending further evidence.

Our findings on secondary outcomes were less conclusive. For cardiovascular mortality, pooled results were not significant, but subgroup analysis revealed a divergence: Observational studies suggested a benefit, whereas the two RCTs did not. This discrepancy is critical and highlights the well-known potential for confounding in observational research, even with statistical adjustment [10]. The open-label design of the included RCTs [5,14] is also a limitation, potentially introducing performance and detection bias, as noted in our quality assessment. The REDUCE-AMI trial [5], a large, contemporary RCT, specifically found no significant difference in the composite endpoint of all-cause mortality or new MI in patients with LVEF ≥ 50% treated with beta-blockers versus no beta-blockers. This contrasts with the all-cause mortality benefit seen in our pooled analysis when including older observational data.

We found no significant impact of beta-blocker therapy on the rates of reinfarction or hospitalization for heart failure or stroke. These findings align with the REDUCE-AMI trial [5] for reinfarction and generally suggest that in a population already benefiting from modern reperfusion and adjunctive therapies, the incremental benefit of prophylactic beta-blockers for these specific endpoints in patients with preserved LVEF may be limited. It is important to consider, however, that beta-blockers might still offer non-mortality benefits not captured in our analysis, such as symptom relief from angina or the prevention of non-fatal arrhythmias.

The substantial heterogeneity observed for all-cause and cardiovascular mortality warrants careful interpretation. Differences in patient populations (e.g., LVEF cutoffs, though all ≥40%), types and doses of beta-blockers used (often unspecified in observational studies), duration of follow-up, and baseline risk profiles across studies likely contributed. Specifically, differences in follow-up duration may have impacted mortality outcomes; shorter follow-up periods may be insufficient to detect a long-term benefit, while longer durations in the modern era may see any benefit attenuated by the high efficacy of other concurrent therapies. Furthermore, patient subgroups not specifically analyzed in this review, such as those with borderline EF (40%-50%), diabetics, or the elderly, may derive differential benefit, but data to explore this were limited. Furthermore, the evolving landscape of STEMI care, with more effective antiplatelet therapies and statins, may have attenuated the relative benefit of beta-blockers over time.

Limitations

This meta-analysis has several key limitations that should be considered when interpreting the findings. First, the evidence base is small, consisting of only two randomized controlled trials (both open-label) and four observational studies. This limited number of RCTs reduces the certainty of the findings and requires reliance on observational data, which are inherently vulnerable to selection bias and unmeasured confounding despite acceptable quality scores.

Second, substantial heterogeneity was observed across several outcomes, reflecting differences in patient populations, study designs, beta-blocker types and dosages, follow-up duration, and outcome definitions. Although random-effects models, subgroup analyses, and sensitivity analyses were performed, this variability could not be fully accounted for and reduces the precision of pooled estimates.

Third, the definition of preserved EF was not consistent across studies. While this review defined HFpEF as LVEF ≥ 40%, some included studies used thresholds of ≥50%, preventing a clean definition of truly preserved EF. Sensitivity analyses excluding the 40%-49% group were not possible due to limited reporting.

Fourth, reporting of missing data was inconsistent across studies, and no imputation could be performed. All analyses relied on available data only, which may lead to over- or underestimation of certain outcomes. Important variables such as beta-blocker dose, type, and adherence were also poorly reported, preventing more granular analysis.

Fifth, variability in reinfarction definitions and non-uniform follow-up durations across studies introduces additional limitations, particularly for comparing long-term mortality. Some outcomes, such as hospitalization for heart failure and stroke, were only reported by the two RCTs, limiting generalizability.

Additionally, a GRADE summary of findings is provided in Table 5, although the overall certainty remains moderate to low due to imprecision and methodological limitations.

**Table 5:** A GRADE-based summary of findings Downgraded for inconsistency: High heterogeneity (I² > 60%) across most primary outcomes, reflecting clinical and methodological variability. Downgraded for indirectness: Ejection fraction (EF) definitions varied slightly across studies; beta-blocker type and dosing were inconsistently reported. Downgraded for imprecision: Wide confidence intervals crossed benefit and harm, especially for cardiovascular mortality, HF hospitalization, and stroke. Downgraded for risk of bias: Observational studies contributed substantially to pooled estimates; randomized controlled trials (RCTs) were open-label. No upgrade for effect size: Although all-cause mortality shows a 40% relative reduction, inconsistency prevents upgrading.

Outcome	No. of Studies (Design)	Participants	Relative Effect (RR, 95% CI)	Absolute Effect	Certainty (GRADE)	Reasons for Downgrading
All-cause mortality	5 (2 RCTs, 3 cohorts)	18,459	RR 0.60 (0.43–0.85)	40 fewer deaths per 1000	Low	High heterogeneity; contribution of observational studies; imprecision
Cardiovascular mortality	4 (2 RCTs, 2 cohorts)	14,784	RR 0.62 (0.28–1.34)	15 fewer deaths per 1000	Very Low	Very wide CI; high heterogeneity; inconsistent effects between RCTs and cohorts
Reinfarction	4 (mixed design)	14,784	RR 0.87 (0.71–1.07)	7 fewer per 1000	Moderate	Downgraded for imprecision
HF hospitalization	2 RCTs	~3000–4000	RR 1.32 (0.60–2.89)	11 more per 1000	Low	Imprecision; moderate heterogeneity
Stroke	2 RCTs	~3000–4000	RR 1.03 (0.53–2.02)	No difference	Low	Imprecision; moderate heterogeneity

Finally, publication and language bias may be present. Non-English studies were excluded, and formal assessment of publication bias could not be performed because fewer than 10 studies were available for each outcome. Most included data were derived from Western populations, and safety outcomes such as bradycardia or hypotension were not assessed.

In summary, while the results are directionally consistent and supported by sensitivity analyses, these limitations highlight the need for larger, well-designed randomized trials in the post-MI HFpEF population to confirm the potential benefits of beta-blockers.

## Conclusions

In patients who have undergone primary PCI for STEMI and have preserved LVEF (≥40%), prophylactic beta-blocker therapy may be associated with a reduction in all-cause mortality. However, while the pooled effect size appears large, the clinical meaningfulness of this finding is uncertain given the significant heterogeneity and its reliance on observational data. Beta-blocker therapy did not demonstrate a consistent benefit in reducing cardiovascular mortality across all study types and did not significantly impact rates of reinfarction or hospitalization for heart failure or stroke. This discrepancy between observational studies and contemporary RCTs suggests the current evidence aligns more with the conservative ESC guidelines rather than the broader AHA/ACC recommendations, and that modifying the latter may be appropriate. This is particularly relevant as ongoing improvements in reperfusion and adjunctive therapies may continue to diminish the incremental benefit of prophylactic beta-blockers.

The existing evidence gap underscores the need for more high-quality, double-blind, placebo-controlled RCTs to definitively clarify the role of this therapy. Future research should not only aim to identify subgroups that might derive particular benefit or harm but also explore the differential effects of beta-blocker type and dose and assess important patient-centered outcomes, such as quality of life and symptom relief, which were not evaluated in the included studies.
